# Flap Reconstruction of the Oropharyngeal Defect After Tumor Resection *via* Combined Transcervical and Transoral Approach in Patients With HPV-Positive and -Negative Oropharyngeal Squamous Cell Carcinoma

**DOI:** 10.3389/fonc.2022.857445

**Published:** 2022-02-24

**Authors:** Jiaming Chen, Jugao Fang, Qi Zhong, Ling Feng, Shizhi He, Hongzhi Ma, Lizhen Hou, Meng Lian, Ru Wang, Xixi Shen, Yifan Yang

**Affiliations:** Department of Otorhinolaryngology Head and Neck Surgery, Beijing Tongren Hospital, Capital Medical University, Beijing, China

**Keywords:** oropharynx, cancer, surgery, approach, parapharyngeal space, prognosis

## Abstract

**Objective:**

To investigate a novel surgical approach of combined transcervical parapharyngeal space (PPS) with the transoral approach to dissect oropharyngeal cancer.

**Methods:**

31 patients who were pathologically diagnosed with oropharyngeal cancer and had undergone surgical treatment in Beijing Tongren Hospital during June 2018 and December 2020 were enrolled. All patients were squamous cell carcinoma patients. There were 25 males and 6 females, and the age ranged between 44 and 70 years old. The number of patients with T1, T2, T3, and T4 stage disease was 8, 15, 8, and 0, respectively, according to the American Joint Committee on Cancer staging method, 8th edition. After the dissection of the submandibular and cervical lymph nodes, the parapharyngeal space was exposed, and the parapharyngeal space lymph node and the outer borderline of the tumor were dissected, and then the inner borderline of the tumor was dissected *via* a transoral approach; the tumor was dissected en bloc, and the defects were reconstructed with the flap from the neck through the parapharyngeal space.

**Results:**

Among the patients enrolled, 21 were HPV positive and 10 were HPV negative. 8 patients were free of lymph node metastasis. The tumor resection margins were negative in all 31 patients. Safe and sufficient excision of tumors was feasible by this new surgical approach, avoiding complications associated with mandibulotomy or lip-splitting. All patients had no obvious dysfunctions of swallowing and voice. By the time of this follow-up, none died caused by OPSCC, and only two patients suffered from local recurrence. The 3-year survival rate is 100%, and the 3-year recurrence-free survival rate is 84.58%.

**Conclusion:**

The surgical approach of combined transcervical parapharyngeal space with the transoral approach was effective and safe. On this basis, this approach has the advantage of fewer postoperative complications and better functional results.

## Introduction

Oropharyngeal cancers occur in the palatine and lingual tonsils, the base of the tongue, the soft palate, and the posterior pharyngeal wall. The most common type is oropharyngeal squamous cell carcinoma (OPSCC). The incidence of OPSCC has increased annually in recent years, especially accompanied by the increase of HPV-related OPSCC (1). The treatment of OPSCC has been updated and improved all the time. Since the 1990s, concurrent chemoradiation therapy (CCRT) has become the standard treatment for OPSCC, especially for the locally advanced OPSCC. Moreover, surgery is often performed for salvage. However, there are many side effects of CCRT. Patients often suffer from acute and late toxicities, which can lead to dysphagia and other dysfunctions (2).

With the rising proportion of HPV-related OPSCC, the onset age is becoming younger, and the treatment of OPSCC now faces new challenges. There is an urgent need to find a new model that can not only guarantee the oncological outcomes but also preserve the oropharyngeal function and reduce the long-term side effect of CCRT. Under this circumstance, radical surgery to reduce the radiotherapy doses has become a promising option for the management of OPSCC. Currently, there are mainly two types of surgical approaches for OPSCC. The one is the transoral approach, which is suitable for the early-stage lesion. The other often concludes the mandibular swing approach which is often chosen for the locally advanced stage OPSCC. No matter what approach, several limitations should be noted. This article will introduce a novel surgical approach of combining transcervical parapharyngeal space and the transoral for OPSCC and assess its effectiveness and functional results.

## Patients and Methods

### Study Population

In this study, the indications for this surgical method were as follows: 1) The T_1-3_ oropharyngeal cancers. 2) The mandible and maxilla were not involved by the tumor. 3) The large vessels (the internal carotid arteries and the internal jugular veins) of the parapharyngeal space were not encapsulated completely by the tumor. 4) Considering the tumor burden and the feasibility of surgery, the metastatic lymph nodes in the parapharyngeal space should be less than 3 cm and the cervical metastatic lymph nodes should be less than 6 cm.

Finally, 31 patients who underwent surgery in Beijing Tongren Hospital between June 2018 and May 2021 were enrolled. The inclusion criteria were patients diagnosed with oropharyngeal squamous cell carcinoma which penetrated to the parapharyngeal space, and who meet the surgical indications and underwent the new surgical approach. The age ranged between 44 and 70 years. All patients had complete clinical data and follow-up information. Individuals who were diagnosed with another confirmed pathological disease or those with incomplete medical records were excluded. Based on the inclusion and exclusion criteria, 31 subjects were eligible for this analysis. Among the 31 patients, their therapeutic schedule was determined after the MDT (multidisciplinary team) meeting. The characteristics of our study population are as shown in **Table 1**.

**Table 1:** Clinical Characteristics of study populations.

Variables	Statistics
**Age in years**	
Min–max	44–70
Mean ± SD	56 ± 7.49
Median	55
**Gender**	
Male	25
Female	6
**Smoking status**	
Yes	13
No	18
**Drinking status**	
Yes	5
No	26
**Primary tumor site**	
Tonsil	21
Soft palate	5
Base of tongue	5
**HPV status**	
Positive	21
Negative	10
**Tumor stage(T)**	
T1	8
T2	15
T3	8
T4	0
**Tumor stage(N)**	
N0	8
N1	12
N2	11
**Clinical stage**	
I–II	21
III–IV	10

In this study, we collected clinical information for each patient, including the patients’ baseline characteristics, postoperative recovery, and pathological examination findings. The VHI-10 and EAT-10 score systems were performed to evaluate the voice and swallowing function respectively 6 months after treatment (3, 4). The study protocol was approved by the Institutional Review Board of Beijing Tongren Hospital of Capital Medical University, and patient approval or informed consent was required for the review of the patients’ medical records.

### Clinical Treatment

#### Surgical Technique

Before the surgery, the enhanced CT and MRI were used routinely to determine the extent of the lesion and invasion of adjacent tissues.

**Figure 1:**
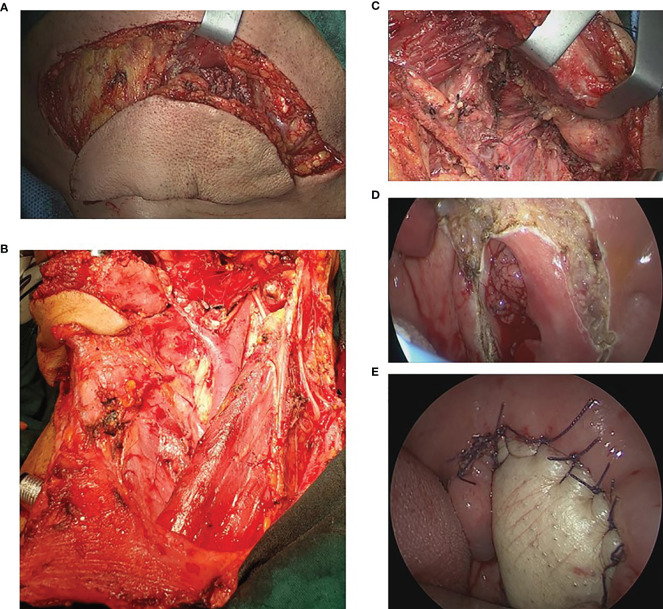
Illustration of the surgical approach. **(A)** The submental island flap was designed and performed. **(B)** The neck dissection was performed. **(C)** After the neck dissection, the parapharyngeal space was exposed. **(D)** The tumor was exposed and dissected *via* the transoral approach. **(E)** The defection was reconstructed with the submental island flap.

There were several fixed steps to finish the surgery through the new approach. After general anesthesia and tracheal intubation, the tumor size and the extent of resection were determined using a direct laryngoscope. For patients whose defects were estimated not suitable for direct saturation, the constructive flap was designed in advance. Usually, the submental island flap and supraclavicular flap were considered firstly. It should be noted that patients choosing the submental island flap need to assess the lymph nodes of Level I. When there were suspicious metastatic lymph nodes in Level I, the submental island flap should not be selected. After the submental island flap was finished, the dissection of cervical lymph nodes can be started (Levels I–IV or Levels I–V). Then, the caudate lobe of the parotid alongside the sternocleidomastoid muscles up to the mastoid was dissected to pull the parotid gland upward, and the digastricus and stylohyoid muscles on the surface of the carotid sheath were cut off. After that, the parapharyngeal space upward along the surface of the carotid sheath was entered. Dissection of the lymph nodes and adipose tissue of the PPS on the surface of the carotid sheath was then performed. Then, the stylopharyngeus muscle was cut off to expose the vessels and nerves in the PPS. It is therefore convenient to find the glossopharyngeal nerve, the hypoglossal nerve, and the accessory nerve above the digastricus muscle. When dissecting the PPS, the surgeons should be careful not to mistake the superior sympathetic ganglion in the rear of the carotid sheath for the lymph node and dissect it. Besides, the posterior pharyngeal space should also be explored. At that time, the outer boundary of the tumor was unveiled. Next, the middle pharyngeal constrictors downward to the hyoid level were explored. If the base of the tongue was suspected to be involved by the tumor before surgery, the lingual nerve should be dissected either. Then, the pharyngeal cavity was entered through the epiglottic vallecula and the lateral pharyngeal wall was dissected. Once the PPS exploration was finished, transoral surgery can be performed. With the help of the Boyle–Davis mouth gag, the oropharynx and the tumor boundary can be visually exposed. The tumor with a 1-cm safety margin was dissected, and then the oral cavity and the PPS were connected directly. After the tumor was resected, the small defect can be closed directly while the defects which cannot be closed directly will need a constructive flap (**Figure 1**).

The drainage tubes were placed in the PPS and cervical region. Preventive tracheotomy was also performed when tumor dissection was finished. 3 days after the surgery, patients can try to block the tracheostomy tube and it can be usually blocked persistently 5–6 days after the surgery. The nasal feeding was usually conserved for 3–5 days.

#### Adjuvant Treatment

According to the suggestion of the radiation experts, patients with T_2_-_3_ lesions and cervical lymph node metastasis should receive postoperative radiotherapy after 4–6 weeks of the surgery. The radiotherapy dose was 55–60 Gy at the surgical region and 50 Gy at the cervical region. Moreover, patients with IV stage tumor and extra-nodal invasion need to receive concurrent radiochemotherapy.

### Statistical Analysis

Statistical analysis was performed using SPSS software. Categorical variables are presented as numbers. Average data were expressed as mean and standard deviation.

## Results

### Postoperative Results

**Figure 2:**
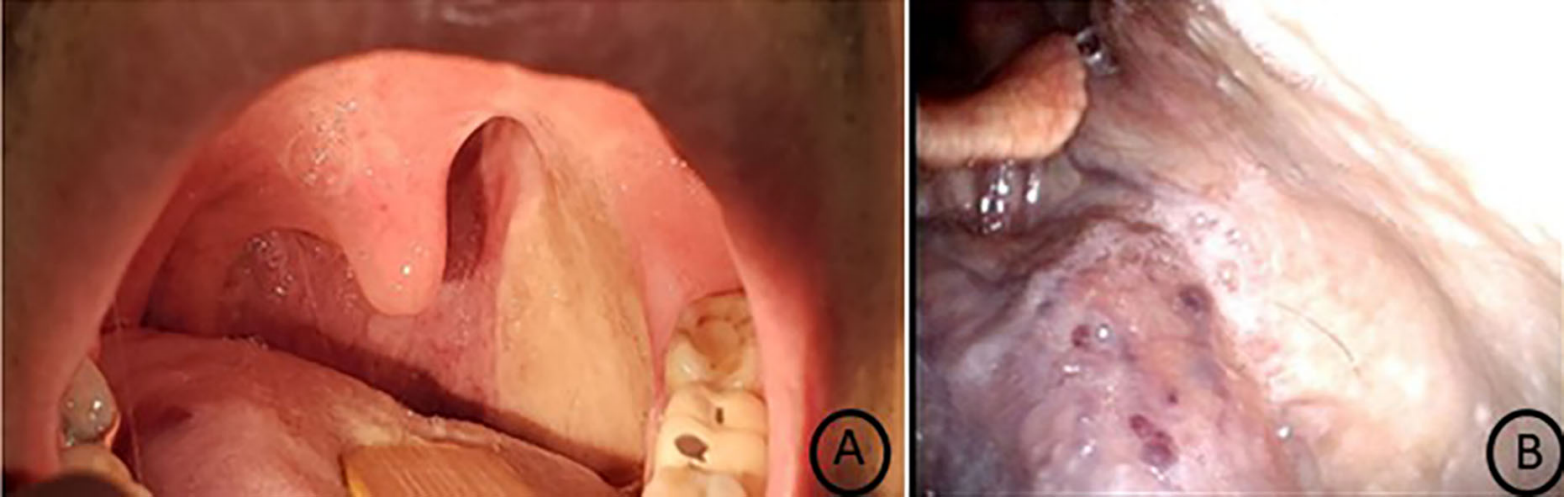
Reexamination 1 year after surgery. **(A)** The reconstructive submental island flap has coalesced with surrounding normal mucosa. **(B)** The pharyngeal wall reconstructed by the submental island flap healed well and had a natural morphology.

**Figure 3:**
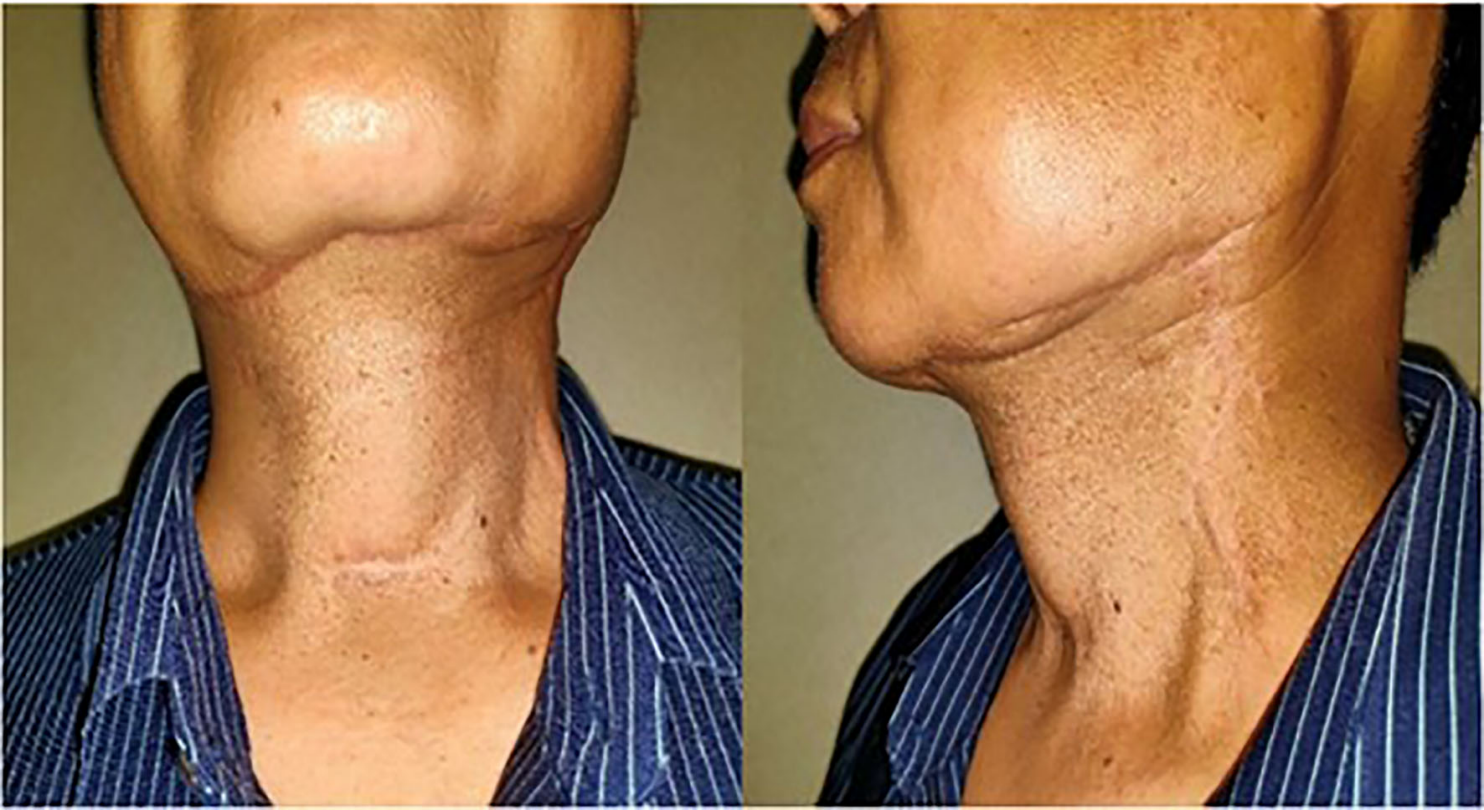
Reexamination 2 years after surgery. There were no obvious facial defects.

31 patients enrolled in this research were all treated with the designed new surgical approach. Postoperative complications occurred in 4 patients (12.9%): two developed pharyngocutaneous fistula (PCF) and recovered after a period of dressing changes. One patient with the submental island flap developed flap necrosis on postoperative day 2 and underwent surgery using the pectoralis major musculocutaneous flap to repair the defect. Another patient developed cervical hemorrhage on postoperative day 5 and also underwent cervical exploratory surgery. Two patients who underwent surgery both recovered. By the end of this follow-up, all patients have removed their tracheostomy tubes. The average time of removing the tracheostomy tube is 20.90 ± 12.35 days after the surgery. The oral intake of food was reintroduced an average of 13.03 ± 6.82 days after surgery (**Figures 2** and **3**).

26 patients with II–IV stages were radiated. Adjuvant chemotherapy (cisplatin) was given to 8 patients. 3 patients were treated with adjuvant targeted therapy (**Table 2**).

**Table 2:** Clinical treatment of study populations.

Variables	Statistics
**Preoperative treatment**	
Induction chemotherapy	10
None	21
**Surgical procedure**	
New surgical approach	31
Lymph node dissection	31
Tracheotomy	31
**Reconstructive flap**	
Submental island flap	20
Pectoralis major musculocutaneous flap	3
Supraclavicular Flap	2
None	6
**Postoperative treatment**	
Radiotherapy	26
Chemotherapy	8
Targeted therapy	3

### Pathological Results

All patients were squamous cell carcinoma patients. Among them, 21 patients were HPV positive (67.74%) and 10 patients were HPV negative. 6 patients had well-differentiated carcinoma, 17 patients had moderately differentiated carcinoma, and 8 patients had poorly differentiated carcinoma. 23 patients were confirmed lymph node metastasis. The most common metastatic region was Level II, with 23 patients (74.19%). Furthermore, one patient had one metastatic lymph node in the parapharyngeal space (metastasis rate 3.2%, 1/31), and another patient had one metastatic lymph node of Level I (metastasis rate 3.2%, 1/31). The tumor resection margins were negative in all 31 patients.

### Survival Outcomes

**Figure 4:**
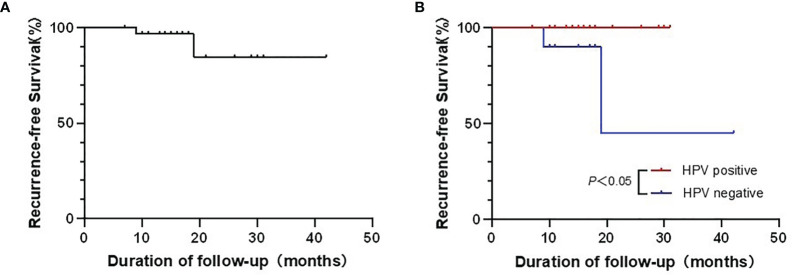
**(A)** The recurrence-free survival (RFS) of this new approach in 31 patients. **(B)** The comparison of RFS between the HPV-positive group and HPV-negative group.

All 31 patients had been followed up postoperatively. By the end of October 26, 2021, this follow-up time, none of the 31 patients died from the disease. Only two patients suffered from local recurrence 9 and 19 months after the surgery, respectively. The two patients were both alive after conservative treatment. The average follow-up after the operation was 16 months, ranging between 41 and 8 months. The median follow-up was 15 months. The 3-year recurrence-free survival is 84.58% (**Figure 4A**). Considering the HPV status, we compared the outcomes of the HPV-positive group and HPV-negative group. The results indicated that the HPV-positive group has a better RFS than the HPV-negative group (*p* < 0.05) (**Figure 4B**).

### Functional Results

The results obtained from the preliminary analysis of functional outcomes are presented in **Table 3**. The VHI-10 score was 2.55 ± 0.95, which means patients undergoing surgery have no severe subjective voice dysfunction. According to the EAT-10 score (2.48 ± 1.16), there is no obvious dysphagia. All patients resumed their preoperative diet.

**Table 3:** Functional outcomes of the study population.

Variables	Statistics (mean ± SD)
VHI-10	2.55 ± 0.95
EAT-10	2.48 ± 1.16

## Discussion

In the past few decades, the treatment of OPSCC has been changed and developed all the time. Until the 1990s, open surgery was the primary choice for OPSCC. However, at that time, the surgical approach which required lip-splitting mandibulotomy or mandible swing often caused severe functional morbidity, especially for speech and swallowing. Therefore, open surgery was largely displaced by concurrent chemoradiotherapy (CCRT) in the 1990s (5). Indeed, CCRT has its own advantages in maintaining the same survival outcomes while protecting patients against the trauma of surgical intervention (6), whereas the long-term complications caused by chemoradiotherapy, such as limited mouth opening and dysphagia, will severely affect patients’ quality of life. In recent years, high-risk human papillomavirus (HPV) is recognized as an important cause of the increasing incidence rates of oropharyngeal squamous cell carcinoma. It was reported that the global range of HPV-attributable fractions (AFs) of OPSCC was between 18.5% and 22.4% (7). However, there is high geographic heterogeneity in AFs of OPSCC, ranging from less than 20% in Southern Europe to more than 60% in North America (8, 9). Moreover, it was estimated that the AFs of OPSCC in China were about 57.6% (10), which was consistent with our data. Unlike HPV-negative OPSCC, OPSCC-related patients tend to be healthier and younger and have significantly improved survival outcomes (1, 11). In this situation, the treatment strategy needs to be changed again, as the proportion of surgery has increased in consideration of the acute and late toxicity caused by CCRT. For the early-stage OPSCC, several recent studies have investigated the survival and functional outcomes comparing the transoral laser microsurgery (TLM) and transoral robotic surgery (TORS) with CCRT. Locoregional control and survival rates in early-stage OPSCC have shown equivalent efficacy. However, the swallowing and voicing function was better in patients who underwent surgery (12–14). These results indicate that TLM and TORS may be the more appropriate choice for early-stage OPSCC.

For the locally advanced OPSCC, the TLM and TORS are apparently not suitable. Even some authors have reported the application of TORS in advanced OPSCC, and most patients are selected with low T stage but advanced cervical disease (15, 16). Open surgery was often performed in advanced OPSCC. For those patients, especially when the tumor was close to or invaded the parapharyngeal space, to achieve adequate visualization, an open approach with lip-splitting mandibulotomy (LSM) is usually necessary. However, significant postoperative complications associated with LSM surgical approaches have been reported, including fixation failure and delayed bone healing (17, 18). When comparing the different surgical methods, a meta-analysis reported that patients who received TORS had better disease-free survival (DFS) and were less likely to need reconstructive flap than other open surgeries (19). Another meta-analysis compared the different surgical methods with and without mandibulotomy, which indicated that the two methods had no difference in overall survival, recurrence-free survival, and postoperative function, but the risk of postoperative complications was significantly reduced in patients who underwent surgery without splitting the mandible (20). These studies suggested that compared to the traditional lip-splitting mandibulotomy (LSM) surgery, new surgical methods such as TORS may leave patients with better postoperative function while ensuring oncological results.

However, the TORS approach has its feedback, too. For tumors close to or involving the parapharyngeal space, the lateral margin may be inadequate and there is a risk of damage to the nerves and great vessels in the parapharyngeal space. Therefore, in this article, we introduced a new surgical approach that combined the transcervical approach passing through the parapharyngeal space combined with the transoral approach. It is applied based on the technique of resecting parapharyngeal cancer through the trans-parotid gland approach. In this way, the surgeon can clearly expose the tumor’s outer boundaries no matter how deep the tumor is. Unlike the traditional LSM, this method does not need to split the mandible while making en bloc resection of the tumor possible, which avoids complications caused by splitting the lip and mandibulotomy. The other advantage of this approach is the clear visualization of the three-dimensional location of the tumor and the great vessels in the PPS. Moreover, it is very easy to finish the dissection of the parapharyngeal space in this way.

As for the reconstruction after the tumor resection, both free and pedicled flaps are feasible. In this study, we recommended the local pedicled flap rather than the free flap for the following reasons. First and foremost, when compared with the free flap, the local pedicled flap has been associated with shorter hospital stays, shorter length of stay in the intensive care unit, and operating time while preserving the functional and oncological outcomes (21, 22). It is because the local pedicled flap does not need the microsurgical anastomosis of the vessels, which can greatly reduce the difficulty of this procedure and make this procedure more suitable for promotion. Furthermore, the pedicled flap, especially the submental island flap, was ideally suited because it satisfied the needs of “thin and soft” for the reconstruction of the oropharyngeal defection. Among the pedicled flaps taken into consideration, the submental island flap was mostly selected. However, it is important to assess the lymph nodes of Level I when using this flap. It has been reported that the rate of occult metastasis of level I lymph nodes was about 10% (23). In this study cohort, we did not find the metastatic lymph nodes when evaluating before the surgery using the CT and ultrasound examination. However, we still dissected the Level I lymph nodes and the submandibular gland. One patient was found to have one metastatic lymph node of Level I. We maintained to make the submental island flap thin enough, and only the skin and subcutaneous tissue of the distal perforator vessels should be reserved for reconstruction. When the submental island flap cannot cover the defect or it is hard to make this flap due to vascular conditions, the supraclavicular flap or the pectoralis major musculocutaneous flap can be used. Besides, we recently performed the free flaps used for reconstruction, such as free radial forearm flaps and medial lower leg flaps. However, these patients were not enrolled in this article for the inadequate follow-up.

On the question of oncologic and functional outcomes, this study found that this approach seems to have a satisfactory result, as none of the 31 patients died from the disease, and only two patients suffered from local recurrence. Indeed, this result may be associated with the short timing of follow-up. Moreover, according to our follow-up findings, patients who underwent this surgery had a decent recovery without obvious dysphonia or dysphagia and did not affect the facial appearance.

To make a balance between controlling the recurrence rate and reducing the radiotherapy response, the selection of radiotherapy dose should be carefully considered. Gido et al. (24) reported that after head and neck tumor resection and flap reconstruction, reducing the radiotherapy dose in the flap area can reduce radiotherapy response while ensuring the radiotherapy effect. Giuseppe et al. (25) also reported that after oral robotic surgical resection of oropharyngeal cancer, the dose of adjuvant radiotherapy was reduced compared with that of concurrent radiotherapy and chemotherapy, and the long-term oncology effect of patients was similar. Therefore, for patients with no high-risk factors and complete tumor resection, the radiotherapy dose is 10 Gy less than the radical dose of radiotherapy without surgery.

Taken together, these results suggest that the surgical approach combining transcervical parapharyngeal space with the transoral approach is safe and effective. There are indeed some inherent flaws. First, the study population is only 31 patients which is a lack of evidence. Second, this research is a retrospective study without enough comparative study. Further studies, which take these variables into account, will need to be undertaken.

## Conclusions

The new surgical approach for OPSCC combined with transcervical parapharyngeal space with the transoral approach could maintain equally therapeutic efficacy with fewer postoperative complications compared to the traditional LSM and reduce the acute and late toxicity caused by CCRT. To thoroughly study this approach, further accumulation of patients is encouraged.

## Data Availability Statement

The original contributions presented in the study are included in the article/supplementary material. Further inquiries can be directed to the corresponding author.

## Ethics Statement

The study protocol was approved by the Institutional Review Board of Beijing Tongren Hospital of Capital Medical University, and patient approval or informed consent was required for the review of the patients’ medical records.

## Author Contributions

JC and JF contributed significantly to the analysis and manuscript preparation. JC and JF performed the data analyses and wrote the manuscript. QZ, LF, SH, HM, LH, ML, RW, XS, and YY helped perform the analysis with constructive discussions. All authors contributed to the article and approved the submitted version.

## Funding

This work was supported by the National Key R&D Program of China (No. 2020YFB1312805), the Beijing Natural Science Foundation Program and Scientific Research Key Program of Beijing Municipal Commission of Education (KZ201910025034), Beijing Municipal Administration of Hospitals’ Ascent Plan (DFL20180202), and Beijing Municipal Administration of Hospitals’ Youth Programme (Code: QMS20210206).

## Conflict of Interest

The authors declare that the research was conducted in the absence of any commercial or financial relationships that could be construed as a potential conflict of interest.

## Publisher’s Note

All claims expressed in this article are solely those of the authors and do not necessarily represent those of their affiliated organizations, or those of the publisher, the editors and the reviewers. Any product that may be evaluated in this article, or claim that may be made by its manufacturer, is not guaranteed or endorsed by the publisher.
